# Most of the tight positional conservation of transcription factor binding sites near the transcription start site reflects their co-localization within regulatory modules

**DOI:** 10.1186/s12859-016-1354-5

**Published:** 2016-11-21

**Authors:** Natalia Acevedo-Luna, Leonardo Mariño-Ramírez, Armand Halbert, Ulla Hansen, David Landsman, John L. Spouge

**Affiliations:** 1 Department of Genetics, Development and Cell Biology, Iowa State University, Ames, IA 50011 USA; 2 National Center for Biotechnology Information, National Library of Medicine, National Institutes of Health, Bethesda, MD 20894 USA; 3 Department of Biology, Boston University, 5 Cummington Mall, Boston, MA 02215 USA

**Keywords:** Transcription factor binding site, Positional preference, Transcription start site

## Abstract

**Background:**

Transcription factors (TFs) form complexes that bind regulatory modules (RMs) within DNA, to control specific sets of genes. Some transcription factor binding sites (TFBSs) near the transcription start site (TSS) display tight positional preferences relative to the TSS. Furthermore, near the TSS, RMs can co-localize TFBSs with each other and the TSS. The proportion of TFBS positional preferences due to TFBS co-localization within RMs is unknown, however. ChIP experiments confirm co-localization of some TFBSs genome-wide, including near the TSS, but they typically examine only a few TFs at a time, using non-physiological conditions that can vary from lab to lab. In contrast, sequence analysis can examine many TFs uniformly and methodically, broadly surveying the co-localization of TFBSs with tight positional preferences relative to the TSS.

**Results:**

Our statistics found 43 significant sets of human motifs in the JASPAR TF Database with positional preferences relative to the TSS, with 38 preferences tight (±5 bp). Each set of motifs corresponded to a gene group of 135 to 3304 genes, with 42/43 (98%) gene groups independently validated by DAVID, a gene ontology database, with FDR < 0.05. Motifs corresponding to two TFBSs in a RM should co-occur more than by chance alone, enriching the intersection of the gene groups corresponding to the two TFs. Thus, a gene-group intersection systematically enriched beyond chance alone provides evidence that the two TFs participate in an RM. Of the 903 = 43*42/2 intersections of the 43 significant gene groups, we found 768/903 (85%) pairs of gene groups with significantly enriched intersections, with 564/768 (73%) intersections independently validated by DAVID with FDR < 0.05. A user-friendly web site at http://go.usa.gov/3kjsH permits biologists to explore the interaction network of our TFBSs to identify candidate subunit RMs.

**Conclusions:**

Gene duplication and convergent evolution within a genome provide obvious biological mechanisms for replicating an RM near the TSS that binds a particular TF subunit. Of all intersections of our 43 significant gene groups, 85% were significantly enriched, with 73% of the significant enrichments independently validated by gene ontology. The co-localization of TFBSs within RMs therefore likely explains much of the tight TFBS positional preferences near the TSS.

**Electronic supplementary material:**

The online version of this article (doi:10.1186/s12859-016-1354-5) contains supplementary material, which is available to authorized users.

## Background

Transcription factors (TFs) form molecular complexes that bind regulatory modules (RMs) within DNA. Many recent experiments attempt to decipher the code for transcription regulation, but despite experimental progress, the molecular code for transcription regulation remains an active area of research. Because in vitro binding experiments do not mimic in vivo concentrations and conditions, computational approaches based solely on sequence data provide reassuring checks on experimental artefacts. In addition, computation is much less expensive than experimentation.

**Fig. 1:**
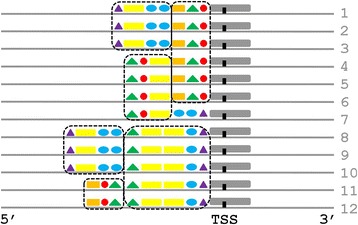
Co-localization of Transcription Factor Binding Sites (TFBSs) within Regulatory Modules (RMs). The DNA coordinate systems near 12 hypothetical genes (*horizontal grey lines*) are shown oriented from 5′ to 3′ on the plus strand, as indicated at the bottom. The genes are aligned according to their TSSs (*vertical black lines*). Within TF molecular complexes during transcription, RNA polymerase II and non-specific TFs (*grey rounded rectangles*) interact with DNA in specific positions relative to the TSS, with different specific TFs interacting with the TFBSs (*colored shapes*) within RMs. Because the RMs have similar structures (*indicated by dotted black rounded rectangles*), their TFBSs have tight positional preferences relative to each other. In other words, the TFBSs must co-localize within the RMs

Molecular complexes of TFs can contain subcomplexes (subunits) that bind to regulatory modules (RMs) in DNA to perform important functions in human gene regulation [1–3]. Experiments often focus on subunits with broad regulatory functions such as non-specific initiation of transcription [4]. Subunits coordinating TF regulation in relatively narrow sets of genes may also be biologically important, but they are probably most studied in experimental systems outside humans (e.g., bacteriophages [5]). In any case, such subunits must interact with similarly structured regulatory modules (RMs) specific to the set of genes. Figure 1 illustrates that to form the RM for each gene, the transcription factor binding sites (TFBSs) within each RM must display tightly consistent positions relative to each other. In other words, the TFBSs must co-localize within the RMs.

Figure 1 illustrates some pertinent features of RMs near the the transcriptional start site (TSS). It is deliberately simplistic in understating the variability of RMs, non-specific TFs, RNA polymerase II, etc. In particular, it does not display at least three important biological complications. First, a gene may have multiple TSSs; second, two TFBSs may overlap; and third, the TBFSs within an RM may not be adjacent. (Because TF subunits are three-dimensional, their contacts with DNA might not always be contiguous.) Nonetheless, Fig. 1 usefully illustrates some consequences of subunits within TF complexes, when the subunits recombine promiscuously in TF complexes like domains in proteins, to coordinate the regulation of specific sets of genes.

Figure 1 illustrates that subunits should influence TFBS positional preferences relative to the TSS near the TSS itself, e.g., the rightmost subunits over lines 1–6 appear in a single position, whereas the leftmost subunits over lines 1–3 and 8–10 appear in two positions. Subunits may also interact with RMs far from the TSS, but intervening subunits may perturb the position of the corresponding RMs relative to the TSS, e.g., the leftmost purple triangles over lines 1–3 and 8–10 appear in two different positions. Thus, tight positional preferences of some TFBSs may reflect their co-localization with each other and with the TSS.

On one hand, experimental results already confirm that some TFBSs have positional preferences. For example, chromatin immunoprecipitation and high-throughput sequencing (ChIP-seq) revealed that many transcription factors have preferred positions and orientations in GC-rich, nucleosome-depleted, and DNase I hypersensitive regions [6]. Similarly, the transcription factor YY1 has distinct activating and repressing functions [7], the specific function depending on position relative to TSS [8]. Moreover, the relative order and exact position of adjacent TFBSs within some RMs determine specific activities within some systems [9] such as the interferon enhanceosome [10].

On the other hand, the extent to which TFBS positional preferences near the TSS reflect co-localization within RMs is unknown. Accordingly, this computational study locates TFBSs near the TSS that have tightly consistent positions relative to each other, initially locating TF motifs with positional preferences relative to the TSS. Some computational studies find TFBSs by identifying statistically overrepresented motifs near proximal promoters [11–14] or with positional preferences [15–18], or both [19, 20]. Because variations in the nucleotide composition near the TSS can complicate finding TFBSs by positional preference, at least one sequence study used a background model accounting for variation of dinucleotide compositions across regulatory regions [21]. The present study therefore identifies TF motifs with positional preferences relative to TSS by combining all three considerations (statistical overrepresentation, positional preference, and oligonucleotide composition) into a single *p*-value described in the Methods section.

By itself, detecting TFBSs with positional preferences relative to the TSS does not imply that the corresponding TFBSs are co-located (i.e., that they have biologically functional positional preferences relative to each other), unless the TFBSs co-regulate the same gene. If they co-regulate, however, the TFBSs co-occur more than they would by chance alone (see Fig. 1; also later, Fig. 5). Thus, the presence of an RM enriches the intersection of the gene groups corresponding to every pair of its TFBSs. Figure 1 illustrates RMs enriching the intersections of gene groups. In Fig. 1, genes 1–6 are all associated with both the rightmost 6 green triangles and rightmost 6 red circles; genes 8–10 are all associated with the leftmost 3 purple triangles and leftmost 3 yellow rectangles. Figure 1 also illustrates that enrichment of gene-group intersections may also occur for pairs of TFBSs in different RMs, but more weakly than for TFBSs in the same RM, e.g., only genes 1–3 are associated with both the 3 rightmost red circles in one RM and 3 purple triangles in another RM.

Thus, for the initial step of detecting TF motifs with positional preferences with respect to the TSS, we collected promoter regions in a block alignment without gaps, with the TSSs aligned in a single column. Our previous studies [17, 22] examined every oligomer of length 8 from the alphabet {*A*, *C*, *G*, *T*} for positional preferences relative to the TSS. In contrast, this study examined every human TF in the JASPAR database [23, 24] to detect sets of TF motifs with a tight positional preference relative to the TSS. Each significant set of motifs corresponded to a group of genes [25, 26]. The web tool for the gene ontology database DAVID (Database for Annotation, Visualization, and Integrated Discovery, Version 6.7, 2010 release) at http://david.abcc.ncifcrf.gov/ validated the biological functionality of each gene group, by using a (modified) Fisher exact test to compare each gene group to gene groups with known biological functions [25–27].

As noted above, the motifs corresponding to two TFBSs co-localized in an RM should co-occur more than by chance alone, i.e., the presence of an RM enriches the intersection of gene groups corresponding to the two TF motifs. To detect enrichment of the intersection of gene groups corresponding to each pair of significant sets of motifs, we performed a right-tailed Fisher exact test.

As described in the Results, Discussion, and Conclusion sections, our statistical results show that in humans, most of the tight positional preferences of TFBSs near the TSS entail co-localization of TFBSs with each other.

## Results

**Fig. 2:**
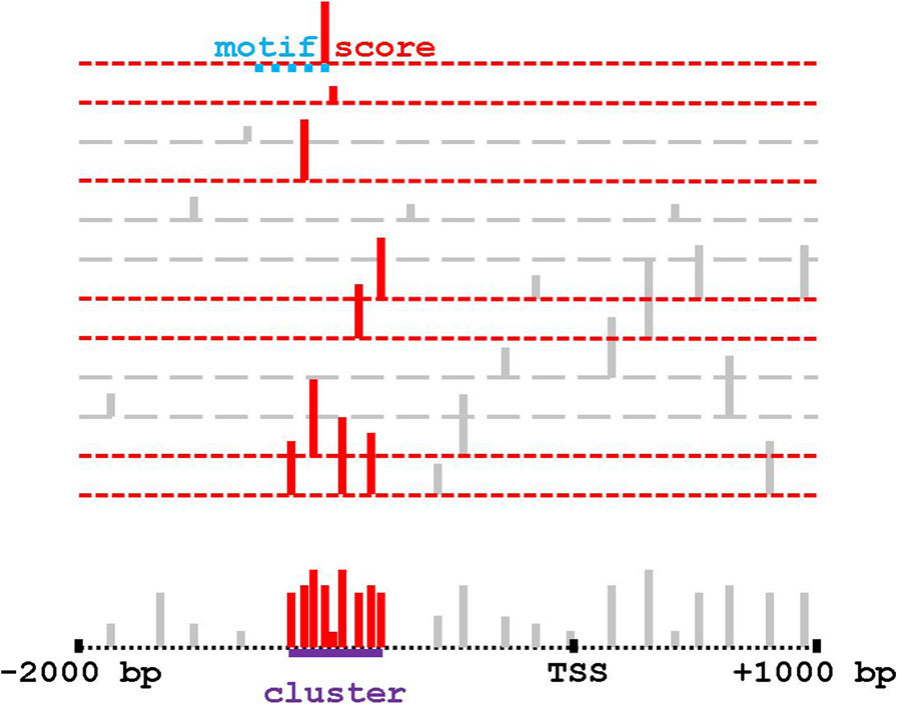
A cluster (a Set of Motifs with a Positional Preference Relative to the TSS). Each *dashed horizontal line* represents a sequence within our Proximal Promoter (PPR) Database, aligned so that all TSSs are in a single column. The *dotted black horizontal line at the bottom* represents the column coordinates within the alignment, running from −2000 bp to +1000 bp on the plus strand, as in Fig. 1. For any fixed TF (e.g., SP1), each *solid vertical line* indicates that the TF’s position-specific scoring matrix has a positive score, corresponding to a subsequence we call a “motif”. Each motif has a motif width, so for computational convenience we assigned the motif’s position and score to its 3′ base (not its 5′ base, as is more common). The top sequence, e.g., displays one motif as a horizontal dotted blue line; and the motif’s positive score, by a vertical solid red line at its 3’ base. Fig. 2 illustrates each positive score twice, once on top of its sequence, and vertically below once again on top of the column coordinates (*dotted black horizontal line*)

Figure 2 illustrates that those motifs with a positional preference relative to the TSS form clusters within the alignment columns. A small *p*-value for a cluster suggests that it contains TFBSs with positional preferences relative to the TSS. The Methods section details the null hypothesis (*H*_0_) of the cluster *p*-value. In its essence, for any given TF, *H*_0_ preserves the number and magnitude of the observed log-odds scores, but distributes them uniformly among the alignment columns. The log-odds scores themselves are the usual logarithm of a ratio, whose numerator is the product of position-specific probabilities (estimated from JASPAR TF count matrices), and whose denominator is a 3rd-order Markov background probability (with transition probabilities re-estimated empirically every 50 bp).

As illustrated in Fig. 2, clusters are sets of TF motifs with statistically significant positional preferences relative to the TSS. To facilitate reproduction of our calculations, the results give all *p*-values without any multiple test correction. We applied statistical tests to the intersections of clusters we considered significant: different *p*-value thresholds would lead to different multiple test corrections. To account for our multiple test protocols, therefore, before giving uncorrected *p*-values, the text also gives the *p*-value thresholds required for a significance level *α* = 0.05 under the Bonferroni correction [28, 29]. In contrast, all validating DAVID *p*-values include DAVID’s Bonferroni correction, thereby accounting directly for the fixed number of biological functions that DAVID examined.

### Validation of the TSS position in the PPR database

Our alignment appeared to anchor the TSS accurately (see Fig. 2), because Figure S1 in the Results section of the Additional file 1 shows an upward spike in TpA composition and a downward spike in transposable element density near the column of the putative TSS.

### Significant clusters and their validation by the DAVID

At *α* = 0.05, the Bonferroni correction for multiple tests on 53 TFs and 2 DNA strands yields a cluster *p*-value threshold *p* = 0.05/(53 × 2) = 4.72 × 10^− 4^. Of the 53 TFs in our JASPAR Database, 21 TFs yielded 43 clusters from the PPR Database significant at *p* ≤ 4.72 × 10^− 4^. In contrast, our negative control with the Random Database (composed of randomly positioned human DNA matched for sequence length and number to our PPR Database) yielded a single cluster with *p* ≤ 0.20 (*p* = 0.14, in fact). The SI describes another negative control with the Random Database with Offsets, which yielded no cluster with *p* ≤ 0.20. At threshold *p* ≤ 0.20, 53 × 2 × 2 = 212 tests yield an expected 212 × 0.2 = 42.4 false positives for a uniformly distributed *p*-value, indicating that with just its single false positive cluster, our cluster *p*-value is extremely conservative.

Table 1 displays the 21 significant clusters on the plus strand; Table 2, the 22 significant clusters on the minus strand. Figure 2 illustrates some terminology in the Tables. As mentioned in Fig. 1, for computational reasons, Fig. 2 assigns position and score to a motif’s 3′ base (not its 5′ base, as is more common). The maximal segment at the bottom of Fig. 2 corresponds to a motif cluster. The left red vertical segment in the cluster corresponds to *Position From* in the Tables; the right, to *Position To*. The cluster’s *spread* is the difference between Position From and Position To plus one. (Thus, e.g., if every motif in a cluster ends in the same alignment column, the cluster has spread 1.) In Fig. 2, the bottom short-dashed red line contributes three motifs to the cluster, so it contains two *multiple motifs*.

**Table 1:** Significant clusters on the plus strand

TF	Cluster *p*-value	DAVID *p*-value	From (bp)	To (bp)	% Multiple motifs
RELA	1.29E-195	1.32E-13	6	9	40
SPI1	1.17E-81	1.01E-09	6	9	1
TFAP2A	3.08E-74	3.86E-12	1	4	21
SP1	8.48E-74	2.97E-08	−78	−36	44
RXRA-VDR	1.89E-58	1.60E-10	12	13	7
RXRA-VDR	2.68E-43	2.98E-08	4	6	5
MYC-MAX	1.20E-34	5.16E-15	1	2	0
NFKB1	1.17E-33	1.83E-04	7	10	28
RORA_2	8.03E-32	3.45E-04	4	5	1
PPARG	7.25E-25	1.25E-06	14	16	3
GABPA	1.90E-23	9.50E-05	7	8	0
SRF	2.56E-18	1.95E-03	2	4	10
NHLH1	1.69E-14	1.04E-06	3	3	0
NHLH1	3.41E-10	7.85E-03	1	1	0
IRF2	4.72E-10	2.44E-05	16	17	7
TAL1-TCF3	9.83E-08	1.58E-04	2	2	0
ELK4	9.80E-07	1.65E-05	8	14	1
STAT1	1.38E-06	1.85E-06	3	4	1
E2F1	4.17E-06	1.27E-01	5	5	0
GABPA	1.78E-04	1.55E-05	−24	−20	2
GABPA	2.08E-04	3.50E-02	1	2	0

**Table 2:** Significant clusters on the minus strand

TF	Cluster *p*-value	DAVID *p*-value	From (bp)	To (bp)	% Multiple motifs
SP1	0.00E + 00	3.39E-12	−106	23	64
RREB1	5.23E-242	1.25E-06	−1	18	82
RELA	1.60E-135	1.12E-12	8	10	35
TFAP2A	6.52E-73	2.31E-09	4	7	24
NFKB1	7.38E-64	1.67E-08	7	11	28
PPARG	1.15E-30	5.52E-06	13	15	3
ETS1	3.94E-27	3.51E-05	7	8	0
TAL1-TCF3	1.43E-17	6.70E-03	4	4	0
MYC-MAX	1.52E-16	2.78E-04	3	3	0
ELK4	1.91E-16	4.79E-03	6	7	0
GABPA	2.89E-16	9.55E-07	7	15	2
FOXF2	5.87E-15	3.59E-04	10	10	0
PAX6	5.05E-13	1.29E-02	4	4	0
NHLH1	1.32E-08	5.07E-03	1	1	0
NHLH1	1.76E-08	1.81E-04	3	3	0
E2F1	1.38E-07	5.38E-06	11	23	12
RXRA-VDR	1.48E-06	3.88E-05	13	13	0
SRF	6.91E-06	7.12E-03	4	6	8
ETS1	8.67E-05	7.56E-04	−3	4	2
TLX1-NFIC	1.05E-04	1.04E-06	10	11	1
MYC-MAX	1.15E-04	4.34E-03	5	5	0
ELK4	2.14E-04	2.57E-08	−27	1	9

For each significant cluster, the Tables give the TF and its (uncorrected) cluster *p*-value. They report the smallest DAVID *p*-value for each cluster, Bonferroni-corrected to account for the number of biological functions that DAVID examined. Because DAVID *p*-values are only for validation, and are therefore already conditional on a multiple-test corrected cluster *p*-value, they require no further multiple-test correction. As indicated in Tables 1 and 2, by being Bonferroni-corrected, the DAVID *p*-value also provides an upper bound on the false discovery rate (FDR). Each of the 43 significant clusters corresponds to a gene group, and DAVID independently validated 42/43 (98%) gene groups with FDR < 0.05 (see Tables 1 and 2).

Figure 1 illustrates that as a typical cluster moves away from the TSS, biological noise should randomly perturb motif positions relative to the TSS, thereby impairing cluster detection. In accord with this expectation, all significant clusters in the PPR Database had “To” positions between −36 and 23 bp, near the TSS.

Eight TFs (E2F1, ETS1, NFKB1, PPARG, RELA, SP1, TAL1-TCF3, and TFAP2A) had two significant clusters; three TFs (ELK4, MYC-MAX, and RXRA-VDR) had three; and two TFs (GABPA and NHLH1) had four. To identify clusters uniquely, we join the TF, Position From, Position To, and the strand (+/−) with colons. Thus, NFKB1:+7:+11:– is the NFKB1 cluster from +7 to +11 bp on the minus strand. Similarly, GABPA:–24:–20:+ is the GABPA cluster from −24 to −20 bp on the plus strand.

The SI describes our measures of TFBS information content, reverse palindromic tendencies, and GC content. The only significant clusters with spreads exceeding 10 bp were E2F1:+11:+23:–, ELK4:–27:+1:–, RREB1:–1:+18:–, SP1:–78:–36:+, and SP1:–106:+23:–. The corresponding transcription factors (E2F1, ELK4, RREB1, and SP1) have neither unusual information content nor unusual reverse palindromic tendencies, but the GC-content of their JASPAR count matrices ranked highly among the TFs studied (SP1 – 1st, E2F1 – 2nd, ELK4 – 8th, and RREB1 – 9th), suggesting their length might be an artefact of the high GC-content in proximal promoters. DAVID *p*-values strongly validated the clusters’ biological functionality, however: 5.38 × 10^−6^ (E2F1:+11:+23:–), 2.57 × 10^−8^ (ELK4:–27:+1:–), 1.25 × 10^−6^ (RREB1:–1:+18:–), 2.97 × 10^−8^ (SP1:–78:–36:+) and 3.39 × 10^−12^ (SP1:–106:+23:–). Thus, although the unusually wide clusters have GC-rich count matrices, they appear biologically functional.

Like composition, tandem repeats can also cause artefactually low cluster *p*-values, because the null hypothesis underlying the cluster *p*-value assumes independent motif positions. To evaluate repetitive artefacts, we examined TF logos in MotifMap [30, 31], but few (if any) displayed obvious periodicities. A sequence with *n* motifs contains *n*–1 multiple motifs that might contribute to repetitive artefacts, however, so beyond DAVID’s validation, we evaluated repetitive artefacts with: (1) the fraction of motifs that were multiple motifs; and (2) cluster spreads (because narrow clusters lessen the opportunity for repetitive artefacts).

Other computations found homotypic clusters in human and other vertebrate genomes for all five significant clusters whose spreads exceeded 10 bp [32, 33]. Experiments also support the biological importance of SP1 homotypic clusters [34]. All five clusters had high multiple motif fractions, between 9 and 82%, consistent with a biological functionality for their homotypic clusters.

Of the remaining 38 significant clusters, only one has a validating DAVID *p*-value *p* > 0.05 (E2F1:5:5:+, with *p* = 0.127). E2F1:5:5:+ has spread 1, so tandem repeats make no contribution to its cluster *p*-value. Tandem repeats are therefore unlikely to have an essential influence on significant clusters having spreads of 10 bp or less.

### The intersection of cluster gene-groups

As illustrated in Fig. 1, biological co-functionality of TFs can enrich the intersection of the corresponding gene groups. Accordingly, the right-tailed Fisher Exact *p*-value tested the 43 * 42 / 2 = 903 intersections of pairs of the cluster gene-groups for enrichment. The left-tailed Fisher Exact Test provided a successful negative control on the right-tailed test: no *p*-value was significant. Surprisingly, however, only one uncorrected left-tailed *p*-value was less than 0.20. The expected number of uniformly distributed *p*-values less than *π* is 903*π*, i.e., 903 × 0.20 ≈ 181 for p ≤ 0.20. The Discussion section and SI conclude, however, that our PPR Database has biases in the genes it contains, artefactually but harmlessly reducing the number of *p*-values *p* ≤ 0.20.

In contrast, the right-tailed *p*-values displayed a full range of values, from 0.00 to 1.00. At *α* = 0.05, the Bonferroni correction for multiple tests involving 903 pairs yields a threshold *p* = 0.05/903 = 5.54 × 10^− 5^. Under the correction, the right-sided Fisher Exact test declared 768/903 (85%) of the cluster-pairs significant at *α* = 0.05. DAVID validated 564/768 (73%) of the significant cluster-pairs with *p* ≤ 0.05.

On theoretical grounds, we suspected that our cluster p-values were very conservative. To verify the suspicion empirically, we examined a superset of the 43 clusters consisting of 66 clusters with an uncorrected cluster *p*-value of *p* ≤ 0.20, to determine the fraction of intersections with significant Fisher p-values and their validation by DAVID. At *α* = 0.05, the Bonferroni correction for multiple tests involving 66 * 65 / 2 = 2145 pairs yields a threshold *p* = 0.05/2145 = 2.33 × 10^− 5^. Under the Bonferroni correction, the right-sided Fisher Exact test declared 1374/2145 (64%) of the cluster-pairs significant at *α* = 0.05. DAVID validated 869/1374 (63%) of the significant cluster-pairs with *p* ≤ 0.05. Thus, the superset of 66 clusters had many intersections with significant Fisher *p*-values validated by DAVID.

**Fig. 3:**
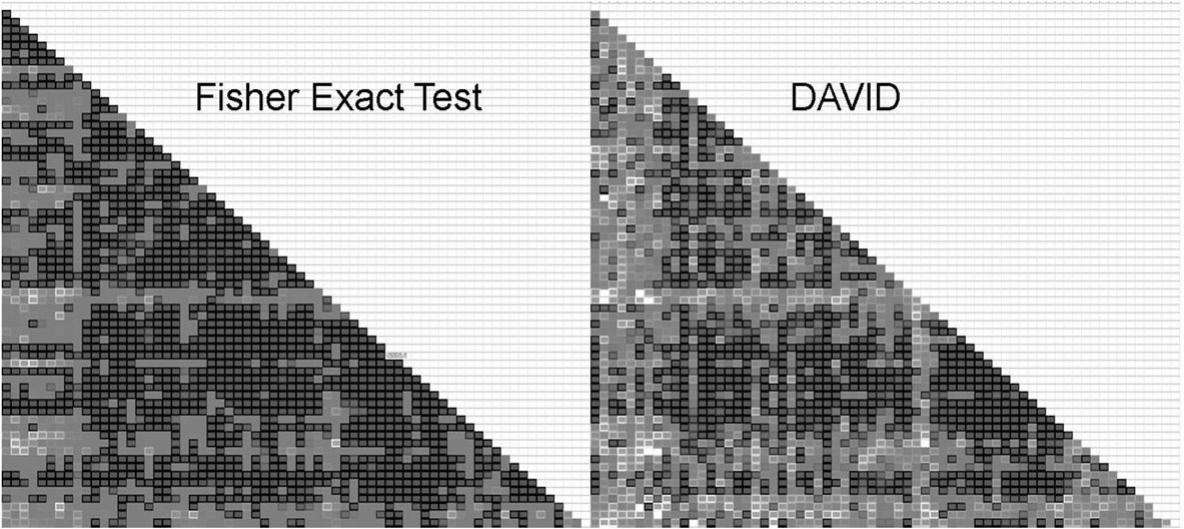
A graphical summary of *p*-values in the SI for gene-group intersections. The two matrices display the *p*-values for the gene-group intersections in graphical form. The matrices correspond to the two tabs in the SI file intersection_p-values.xlsx (although http://go.usa.gov/3kjsH displays the *p*-values more conveniently). The matrices omit their upper triangle, because they are symmetric. They also omit their diagonal, because it corresponds to the intersections of each gene group with itself. In each matrix, each of the 66 (unlabeled) rows corresponds to a single gene group in the SI. In the SI and in Fig. 3, each gene-group corresponds to an uncorrected cluster *p*-value *p* ≤ 0.20. (In contrast, the present, main article confines itself to examining clusters significant at *α* = 0.05 with *p* ≤ 4.72 × 10^− 4^.) The row order in each matrix follows the SI, where the sort is primarily alphabetical on the TF and then on the *p*-value. The magnitudes of the Fisher Exact *p*-values for the gene-group intersections are in shades of gray: *black* indicates 0.0; white, 1.0; and 50% gray, the threshold for significance at *α* = 0.05. For the Fisher Exact Test *p*-values, the threshold is *p* = 2.77 × 10^− 5^ after the present article’s multiple correction; for validating DAVID *p*-values, *p* = 0.05. The font for the (unreadable) *p*-values is 50% gray, making gray on black significant; gray on white, not significant; 50% gray, borderline significant, etc. Each of the 12 white entries in the DAVID matrix indicates a *p*-value that DAVID censored because the gene-group intersection was too small

Figure 3 summarizes qualitatively the patterns of significance and validation for the superset, given in full in Additional file 2. To aid experimental biologists in examining results for particular TFs, however, our results are available on the Web in a user-friendly form at http://go.usa.gov/3kjsH. As noted above, our cluster *p*-value is extremely conservative, and validation with DAVID *p*-values indicates that even some clusters with *p* ≈ 0.20 have biological functions. The file intersection_p-values.xlsx in the SI therefore contains a complete table of right-sided Fisher Exact *p*-values for all clusters whose uncorrected cluster *p*-value *p* ≤ 0.20. Because of their number, the Discussion section can examine only a few intersections explicitly.

## Discussion

A subunit within a TF complex interacts with DNA at an RM containing TFBSs with tight positional preferences with respect to one another (see Fig. 1). If the RM has a positional preference relative to the TSS, the preference propagates to the TFBSs in the RM. Some TFBSs have tight positional preferences relative to the TSS, but the fraction of these TFBSs associated with co-localization in RMs is unknown. Our methods found 43 significant sets of TF motifs with positional preferences relative to the TSS (“motif clusters”, see Fig. 2). Only five clusters lacked a tight positional preference (±5 bp). Note that a statistical method using broad bins [20] would likely be unsuitable for detecting an RM with tight positional preferences.

Each motif cluster corresponded to a group of 135 to 3304 genes, so each gene group contained 2.3 to 56.6% of the 5834 genes in our PPR Database. The corresponding numbers for tight clusters were 135 to 1696 genes (2.3 to 29.1%). When predicting TFBSs with TF motifs, false positives are more common than false negatives. Moreover, the Methods subsection, “*A Cluster Probably Includes Most TFBSs within Its Columns as Motifs*,” shows that false negatives are likely rare within motif clusters. The percentages given are therefore larger than their probable true values, justifying calling at least some gene groups a “specific set of genes”.

Tight positional preferences relative to the TSS do not imply that two TFBSs have a biologically functional tight positional preference relative to each other, unless the TFBSs co-regulate the same gene. As Figs. 1 and 5 illustrate, motifs corresponding to two TFs in an RM should co-occur more than by chance alone, however, enriching the intersections of the corresponding gene groups. Thus, a gene-group intersection systematically enriched beyond chance alone provides evidence that the two TFs participate in an RM. Using the 43 gene groups corresponding to the 43 significant clusters, a right-tailed Fisher Exact test found that 768 pairs of gene groups had significantly enriched intersections.

An analysis of gene ontology using DAVID validated the biological functionality of many significant gene groups, sometimes with false discovery rates less than 10^−4^. In addition, DAVID validated many intersections of gene groups. On one hand, some TF studies have validated their results with the same experimental TF sites that contributed to the count matrices used for discovery. In contrast, DAVID *p*-values validated significant clusters and their intersections, making validation here independent of discovery.

The results in the present computational study therefore incidentally (and unsurprisingly) support the existence near the TSS of RMs coordinating the regulation of specific sets of genes. Gene duplication and convergent evolution provide obvious biological mechanisms for generating the RMs. To facilitate further experimental discovery of such RMs, biologists can mine the user-friendly interface at http://go.usa.gov/3kjsH, to trace TF interactions corresponding to significantly enriched intersections and thereby to discover candidate RMs.

Many computer programs predict TFBSs (reviewed by [35]). Some programs focus on sequence pattern (P-Match [36]; SiTaR [37]), particularly early programs (reviewed in [38]). Several exploit combinations of motifs, but not consistent positioning (Cister [39]; COMET [40]; AliBaba2 [41]; Ahab [42–44]; SCORE [45]). Programs based on Hidden Markov models can discover tightly organized RMs, but few such programs exist (EMCMODULE, [46]). Instead, most newer programs combine phylogeny and possibly other information with TFBS patterns, either with consistent positioning (Stubb [47]; EMMA [48]; TWINE [49]) or without it ([50]; PhyloCon [51]; CisPlusFinder [52]; cisTargetX [53, 54]). Programs searching a single genome for the consistent positional preferences within RMs are therefore surprisingly rare [55].

Notably, all our significant clusters occurred within about 40 bp of the TSS. The absence of significant clusters distant from the TSS tends to deny the existence of RMs distant from the TSS but with tight positional preferences relative to it. (Note, however, that our study uses DNA sequence only, so DNA structural preferences relative to the TSS remain a possibility in three-dimensions).

The 43 significant clusters yielded 768 pairs of gene groups with significantly enriched intersections. The present article cannot examine every significant intersection, but the narrow, scattered results below suggest that specialists might find results in the SI and at the URL http://go.usa.gov/3kjsH interesting. Although the artefact in the previous paragraph influences left-sided Fisher exact *p*-values, DAVID validation of gene-group intersections indicates that the artefact had no essential effect on the right-sided Fisher exact *p*-values.

At http://go.usa.gov/3kjsH, by clicking radio buttons for ETS1 and GABPA, and then clicking “Submit”, we find that ETS1:+7:+8:– has cluster and validating DAVID *p*-values of 3.94 × 10^−27^ and 3.51 × 10^−5^; GABPA:+7:+8:+, of 1.90 × 10^−23^ and 9.50 × 10^−5^; their intersection, Fisher Exact and validating DAVID *p*-values of 1.91 × 10^−164^ and 6.56 × 10^−3^. The extraordinarily small p-values indicate with remarkable surety that: (1) the two TF motif clusters ETS1:+7:+8:– and GABPA:+7:+8:+ correspond to TFBS clusters; and (2) the bidirectional TFBS clusters interact biologically. In fact, the literature confirms the conclusions. GABPA redundantly occupies ETS1 TFBSs in promoters of housekeeping genes, whereas ETS1 specifically occupies the ETS1 TFBSs in enhancers of T cell-specific genes [56]. Moreover, a p53 mutant preferred binding to the bidirectional promoters if several ETS1 and GABPA TFs were bound nearby [57]. Interestingly, GABPA has another significant cluster on the opposite strand near GABPA:+7:+8:+: GABPA:+7:+15:– has cluster and validating DAVID *p*-values of 4.83 × 10^−4^ and 1.06 × 10^−5^; the intersection of GABPA:+7:+15:– and ETS1:+7:+8:–, Fisher Exact and validating DAVID *p*-values of 4.83 × 10^−4^ and 1.06 × 10^−5^. Thus, the unidirectional pair GABPA:+7:+15:– and ETS1:+7:+8:–, though much less striking than the bidirectional pair previously mentioned and apparently unknown, probably also has biological functions.

SP1 motifs provide general transcription signals near TSSs. Indeed, a colored table in the SI highlights their enriched intersections with many other TF motif clusters, graphically displaying the striking promiscuity of the two SP1 motif clusters in Tables 1 and 2. Our statistical methods tuned their single adjustable parameter, so that most significant motif clusters had tight positional preferences relative to the TSS (±5 bp). The absence of broad bins (e.g., a window size of 31, as in [20]) suggests that motif clusters like the SP1 clusters, whose spreads are unusually broad (e.g., about 100 bp), have biological functions distinctly different from participation in an RM [58].

At http://go.usa.gov/3kjsH, by clicking radio buttons for Sp1 and E2F1, and later Sp1 and ETS1, we find that the Sp1 clusters had several significant intersections with both E2F1 and ETS1. Experiments supported E2F-Sp1 interactions near: dihydrofolate reductase in Chinese hamster [59], dihydrofolate reductase in human osteosarcoma [60], fibroblast CTP:phosphocholine cytidylyltransferase in mouse embryo [61], thymidine kinase in mouse [62], RIP140 in human [63], CDKN2A [64], HMGA1 [65], MYCN [66], and CDKN2C [67]. They also supported ETS-Sp1 interactions near the Runx2 P1 promoter [68], PAI-1 [69], ITGA11 [70], and Npr1 [71].

## Conclusions

Our statistics found 43 significant sets of human motifs in the JASPAR TF Database with positional preferences relative to the TSS, with 38 preferences tight (±5 bp). Each set of motifs corresponds to a group of genes. Of all intersections of these 43 significant gene groups, 768/(43*42/2) ≈ 85% were significantly enriched with 564/768 (73%) intersections independently validated by DAVID with FDR < 0.05. The co-localization of TFBSs within RMs therefore likely explains much of the tight TFBS positional preferences near the TSS.

## Methods (Fig. 4)

**Fig. 4:**
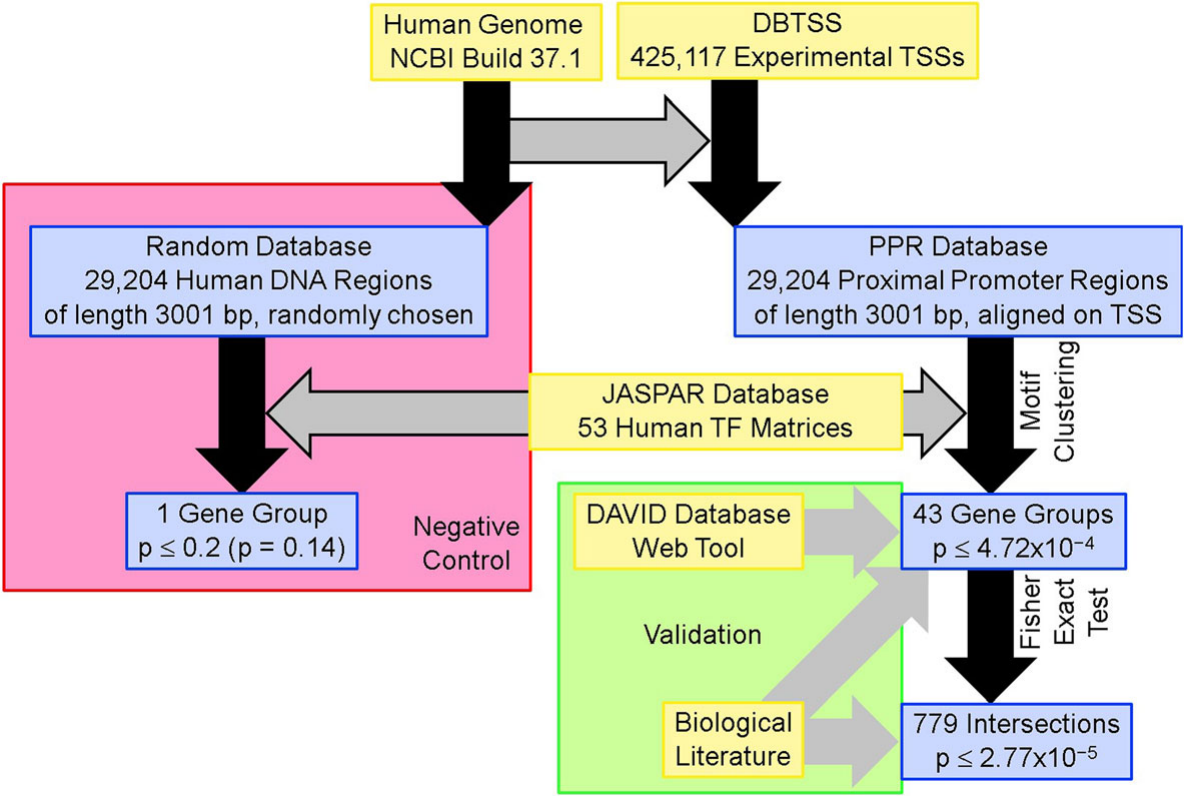
An overview of the workflow in the methods. Primary data sources appear as yellow boxes; the derived data, as blue boxes. The *black arrows* indicate the primary workflow, with *bordered grey arrows* indicating ancillary contributions. The pink box contains the workflow for the primary negative control; the green box, the validation workflow, with its grey arrows indicating validation steps. All *p*-values shown are uncorrected. The *p*-values on the right all retained significance at *α* = 0.05 against multiple-test corrections. The Materials and Methods section gives details

### The PPR and random databases

The publicly available Database of Transcriptional Start Sites (DBTSS) [72] provided about 1.8 million experimentally characterized 5′-end clones from full-length human cDNAs. The experimental clones corresponded to 425,117 transcription start sites (TSSs). Because of alternative TSSs, the experimental TSSs corresponded to 14,628 human RefSeq [73] genes. Our “PPR Database” of proximal promoter regions (PPRs) initially contained every RefSeq TSS within ±1000 bp of the start of an annotated RefSeq gene transcript. If several RefSeq TSSs were within ±1000 bp of the same start, we discarded all but the RefSeq TSS closest to the experimental TSS. Henceforth, “TSS” refers solely to the remaining RefSeq TSS. We aligned the corresponding PPRs in DBTSS to the human genome (NCBI build 37.1).

Standard nomenclature designates the two strands as “plus” (non-template) and “minus” (template). In the following, coordinates in bp correspond to the numbering on the plus strand, with positive bp indicating the 3′ direction from the TSS; negative bp, the 5′ direction. The standard coordinate system places the TSS at +1 bp; the next base in the 5′ direction on the plus strand, at −1 bp.

As in previous studies [22], if a PPR mapped unambiguously, we extended it to include 3001 bp, with coordinates −2000 to −1 bp and +1 to +1001 bp; otherwise, we discarded the PPR [14]. We also discarded replicate sequences and sequences containing nucleotides outside the unambiguous alphabet {*A*, *C*, *G*, *T*}, leaving 29,204 sequences.

We formed a (gapless) block alignment by anchoring the 3001 bp of each of the 29,204 PPR sequences on the corresponding TSS, i.e., we placed each putative TSS in alignment column 2000. After discounting alternative TSSs and alternative splices, the 29,204 PPR sequences corresponded to 5834 distinct genes. As a negative control, we also extracted 29,204 sequences from the human genome (NCBI, build 37.1), one for each of the sequences in the PPR Database. Chosen independently and uniformly at random, each sequence had length 3001 bp. The corresponding 29,204 random sequences constituted our “Random Database”.

The PPR Database contained 29,204 sequences but only 5834 genes, so many of its sequences overlapped with each other. Although the Random Database does not control for overlaps in the PPR sequences, the Additional file 1 describes an extra, unusually elaborate negative control, the “Random Database with Offsets”, which we constructed to rule out spuriously low *p*-values due to sequence overlaps.

### JASPAR count matrices

We then extracted 53 count matrices labelled “species Homo sapiens” from the JASPAR database of transcription factor binding sites [23, 24]. The SI details the following calculations, which we performed for each of the 53 TF matrices from JASPAR.

### Local sum statistic for detecting TFBSs with positional preferences

At each position within each sequence of the PPR Database, we calculated a log-odds score for the presence of a TF motif. For the null hypothesis, we re-estimated background probabilities every 23 columns from a 3rd-order background Markov model fitted to a window of length 50 = 23 + 21 + 3 + 3. The number 23 is the difference between 50 (a nice, round but otherwise arbitrary number) and a sum corresponding to the maximum length (21) of a JASPAR count matrix [IRF1 or REST], plus the letter-triples before (3) and after (3) a putative site. The letter-triples are required to calculate the site probability under a 3rd-order Markov model accounting for sequence context on both sides of a site. (The SI describes the mathematics of the “context-2 model” [74]). Now, fix the TF under discussion. For the alternative hypothesis of a TFBS, we calculated model probabilities from the JASPAR count matrix for the TF and a non-informative Dirichlet prior (pseudo-count 0.5 for every nucleotide). The sum of the log-odds scores within each column of the block alignment scored the column for the presence of the TF motif. Negative sums were ignored by setting them to 0, yielding scores *x*_*i*_ > 0.

For consistency with previous notations, let the segment (*i*, *j*] denote the integer subset {*k* : *i* < *k* ≤ *j*}. Additionally, let *g* be an arbitrary parameter (to be determined later). Given the “global sum” *S*_*i*_ = ∑_*j* = 1_^*i*^(*x*_*j*_ − *g*) = ∑_*j* = 1_^*i*^ *x*_*j*_ − *ig*, define the “segmental sum” *S*_(*i*,*j*]_ = *S*_*j*_ − *S*_*i*_ for each segment, and the “local sum” *Ŝ* = max_0 ≤ *i* ≤ *j*_ *S*_(*i*,*j*]_ for each alignment column *j*. Others note analogies between local sums and the BLAST statistic in sequence alignment [75], so we call *g* a “gap penalty”. The Ruzzo-Tompa algorithm calculates maximal segments (*i*, *j*] [75]. The maximal segments, which satisfy *S*_(*i*,*j*]_ = *Ŝ*_*j*_ > 0, yield contiguous alignment columns rich in motifs [76, 77]. (See Fig. 2.) Karlin-Altschul statistics [78] provide *p*-values to evaluate the statistical significance of the local scores *Ŝ*_*j*_ = *S*_(*i*,*j*]_ corresponding to the maximal segments (*i*, *j*] [40].

The motifs contributing to each maximal segment (*i*, *j*] therefore form a “(motif) cluster” whose score equals the sum of the contributing motif scores. (See Fig. 2.) The motifs in the cluster determine a “gene group”. In Fig. 2, e.g., each motif in the maximal segment corresponds to a gene, namely, the sequences corresponding to short-dashed red lines.

### A cluster probably includes most TFBSs within its columns as motifs

This assertion fits into the flow of the discourse here, although it is important only in the Discussion section. By definition, a TF motif is a subsequence with positive score *x*_*i*_ > 0. The cluster therefore includes every TFBS with a positive score *x*_*i*_ > 0 within its columns (see Fig. 2.). The log-odds scores *x*_*i*_ derive from JASPAR count matrices; the matrices themselves derive from experimental TFBSs. Thus, each TFBS has a positive score *x*_*i*_ > 0, unless it has a sequence pattern inconsistent with other, experimentally derived TFBSs. By definition, such inconsistency is rare, whenever most TFBSs have a consistent sequence pattern. Consequently, most genes (within the PPR Dataset) regulated by TFBSs at positions corresponding to a cluster contribute motifs to the cluster.

### Choice of the gap penalty *g*

Thus far, *g* has been arbitrary. Now, for each TF, we normalize *g* by the TF’s average score per column $\overline{s}={n}^{-1}{S}_n$. Thus, $g=\rho \overline{s}$, where the factor $\overline{s}$ is TF-specific, but all TFs share the arbitrary parameter *ρ*. The normalized gap penalty *ρ* then controls the spread of all TF clusters simultaneously, as follows. As in local alignment [79, 80], extreme-value statistics pertain in a logarithmic regime (here, detailed calculations show that the logarithmic regime corresponds to *ρ* > 1) [81–83]. Moreover, the cluster spreads decrease as *ρ* increases (a phenomenon analogous to alignment lengths decreasing as the alignment gap penalty increases). In accord with the biological aims expressed in the legend of Fig. 1, to infer that a typical significant cluster corresponds to the tight positional preference of a TFBS within a RM, the typical cluster spread should be no more than (say) 10 bp (i.e., ±5 bp). Empirically, we found that such spreads corresponded to a normalized gap penalty of about *ρ* = 1.4. The SI details the exploratory process leading to *ρ* = 1.4. The resulting clusters were relatively robust against perturbations *ρ* = 1.4 ± 0.1.

### DAVID web tool for evaluating the biological function of a group of genes

The DAVID Web Tool Version 6.7 (2010 release) at http://david.abcc.ncifcrf.gov/ provides a modified Fisher exact test to validate the biological functionality of a gene group by comparing the gene group to gene groups with known biological functions [25–27]. Our “DAVID Dataset” represented each cluster’s gene group as a set of RefSeq NP numbers, uniqued so that each gene corresponded to exactly one RefSeq NP.

**Fig. 5:**
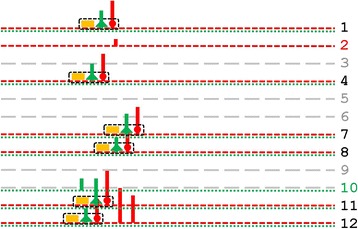
An RM enriches the intersection of two gene groups. A hypothetical RM (*indicated by dotted black rounded rectangles*) is illustrated, similar to an RM in Fig. 1. The RM contains three TFBSs, corresponding to an orange rectangle, a green triangle, and a red circle. The “red circular TF” yields motif scores (*vertical red lines*) corresponding to the maximal segment and motif cluster shown in Fig. 2. Like the red circular TF, the green triangular TF yields its own motif scores (*vertical green lines*). Not every “red” or “green” motif corresponds to a TFBS in the RM, but nonetheless, the RM enriches the intersection of the two cluster gene groups (*horizontal lines, either red dashed or green dotted*)

The genes in DBTSS have biases (e.g., expression) that could propagate to the PPR Database and thence to our DAVID Dataset. To mitigate biases, therefore, we used the DAVID Dataset (and not the full complement of human genes) as the universe of genes under consideration when: (1) DAVID assessed our clusters’ functionality, and (2) the Fisher Exact test assessed the enrichment of the intersections of cluster gene-groups. (See Fig. 5.)

### Fisher exact tests of intersections of cluster gene-groups

A right-tailed Fisher Exact test assessed whether pairs of cluster gene-groups had an unusually large intersection, suggesting possible enrichment by an RM (see Fig. 5). We used the left-tailed test as a negative control (since we did not expect the left-tailed test to yield statistical significance). The Results section describes our 43 significant clusters. Because their 43 × 42/2 = 903 intersections are so numerous, we relegate the complete report of their right-tailed Fisher Exact and their DAVID *p*-values (as described above) to the SI. The *p*-values are also available through our user-friendly interface at http://go.usa.gov/3kjsH. To achieve significance level *α* = 0.05, the Fisher Exact tests needed to yield p ≤ 0.05/(2 × 903) = 2.77 × 10^− 5^.

## Additional files

Additional file 1: Contains supplementary Methods, Results, and Discussion. (DOCX 448 kb)
Additional file 2: Contains the intersection p-values diagrammed in Fig. 3. (XLSX 78 kb)

## Abbreviations

PPR: Proximal promoter
RM: Regulatory module
TF: Transcription factor
TFBS: Transcription factor binding site
TSS: Transcription start site
