# Supplementary material for: Most of the tight positional conservation of transcription factor binding sites near the transcription start site reflects their co-localization within regulatory modules
Source: BMC Bioinformatics. 2016 Nov 21;17:479. doi: 10.1186/s12859-016-1354-5 (PMC5117513; doi:10.1186/s12859-016-1354-5)
Supplement: Additional file 1: — Contains supplementary Methods, Results, and Discussion. (DOCX 448 kb) [file 12859_2016_1354_MOESM1_ESM.docx]

# System and Methods

**The Random Database with Offsets:** We constructed a negative control secondary to the Random Database as follows, to ensure that correlations from overlapping PPR sequences did not cause any spuriously low p-values. Each sequence in the PPR Database corresponded to a unique genomic position within NCBI build 37.1. For each 3001 bp sequence, the PPR Database recorded the chromosomal region and the sequence start. If two TSSs were from the same genomic region, we calculated their “offset”, the difference of their TSS coordinates.

As a specific example from the PPR Database, in region 1p22-p21, ENTREZGENE 5825 (ATP-binding cassette, sub-family D (ALD), member 3) started at 7409; ENTREZGENE 1266 (calponin 3, acidic), at 7815. Their offset is 7815-7409 = 406, suggesting possibly that the two PPR sequences might be the same sequence from region 1p22-p21, offset by 406 bp.

The PPR Database contained 1745 distinct chromosomal regions, so we chose independently and uniformly at random, 1745 sequences of length 3001 bp from the human genome (NCBI, build 37.1). To match the offsets in the PPR Database (described above), we then replicated and circularly permuted the random sequences as necessary. (The circular permutation eases programming, because it mimics sequence overlaps in the PPR Database, while requiring only random genomic sequences of fixed length 3001.) The resulting 29,204 sequences constituted our “Random Database with Offsets”.

As an irritating complication, some of the regions in the PPR Database contained more than a single gene coordinate system. Within 610 chromosomal regions, e.g., 785 pairs of coordinates were identical but in fact corresponded to distinct PPR sequences. The sequences in the Random Database with Offsets therefore had more sequence overlaps than the PPR Database, but the extra overlaps only strengthen (not weaken) the negative control.

**The Calculation of a Log-odds Score for Inferring TFBSs within the PPR Database:** In the following, $l$ (possibly adorned with subscripts, etc.) represents a letter from the unambiguous nucleotide alphabet $A=\left\{A,C,G,T\right\}$; $w$, a nucleotide word composed of letters, e.g. TGGA. First, we explain our methods with general parameter values (e.g., general Markov model order $\omega$), before selecting particular parameter values and probability estimates (e.g., before selecting $\omega=3$). We applied the methods below separately, to both minus and plus DNA strands. The relevant log-odds score uses the following null and alternative hypotheses.

Consider a sequence of length $n$, denoted $\mathbf{a}:=\left(a_{0},...,a_{n-1}\right)$. Under the null hypothesis $H_{0}$, which excludes a TFBS, let $\mathbf{a}$ be generated by a background Markov model of order $\omega$, where the Markov state-space $W$ consists of all nucleotide words $w=l_{1}l_{2}...l_{\omega}$ of length $\omega$, where the transition probability from $w$ to $w'=l'_{1}l'_{2}...l'_{\omega}$ is denoted by $p\left(w,w'\right)$. (Thus, $p\left(w,w'\right)=0$ for $w'=l'_{1}l'_{2}...l'_{\omega}$ unless $l'_{i}=l_{i+1}$ for $i=1,...,\omega-1$.) The random physical processes generating the DNA sequence are assumed symmetric with respect to sequence orientation, making the Markov chain reversible, so its (unique) equilibrium probability distribution $\left\{\pi\left(w\right)\right\}$ satisfies $\pi\left(w\right)p\left(w,w'\right)=\pi\left(w'\right)p\left(w',w\right)$. Denote probability and expectation under the null hypothesis by and . Under $H_{0}$, the random sequence $\mathbf{A}$ equals $\mathbf{a}$ with probability

,

where $w_{k}=a_{k}...a_{k+\omega-1}\in A^{\omega}$.

Consider now a TFBS of length $t$, where the $k$-th letter ($k=0,...,t-1$) in a random site is $a\in A$ with probability $q_{k}\left(a\right)$ ($k=0,...,t-1$). Under the alternative hypothesis $H_{1}$, let a TFBS be inserted between two equilibrium Markov sequences of length $J$ and $n-\left(J+t\right)$, where the starting position $J$ is chosen uniformly at random from $\left\{0,...,n-1\right\}$. Because of superior empirical performance [[1](#_ENREF_1)], we preferred this model, the so-called “context-2” Markov model, to other Markov models extant in the bioinformatics literature [[2](#_ENREF_2), [3](#_ENREF_3)].

Assume the random TF motif and its bordering sequences are independent. Denote probability and expectation under $H_{1}$ by and . Under the alternative hypothesis, the random sequence $\mathbf{A}$ equals $\mathbf{a}$ and contains a TF motif at the $J=j$-th position with probability

,

where $\left(n-t\right)^{-1}$ accounts for the uniform random placement of the TF motif. The corresponding odds-ratio is

.

**Local Score for Inferring TFBSs Clustered in Alignment Columns:** Consider the $S$ = 29,204 sequences $\mathbf{a}^{\left(s\right)}$ ($s=1,...,S$) in our PPR Database, each with length $n=3001$. Each position $j$ in each sequence $\mathbf{a}^{\left(s\right)}$ corresponds to a log odds-ratio $x\left(j,s\right)=\log r\left(j,\mathbf{a}^{\left(s\right)}\right)$. For each column $j=0,....,3000$ in the anchored alignment, define the column score $x'_{j}=\sum_{s=1}^{S} x\left(j,s\right)$, the total of the scores $x\left(j,s\right)$ in column $j$. Truncate the column totals $x'_{j}$ at 0, to yield the positive totals $x_{j}=x'_{j}$ if $x'_{j}>0$, and $x_{j}=0$ otherwise. Let the numerical sequence $\left(x_{i}:i=1,2,...,n\right)$ contain $n'$ positive totals $\left\{x_{n\left(i\right)}:i=1,2,...,n'\right\}$.

**The Implementation of a Windowed Markov Background Model:** The calculation of a Markov background probability for the log odds-ratio $r\left(j,\mathbf{a}^{\left(s\right)}\right)$ follows.

For a Markov model of order $\omega$, Markov states correspond to words $w=l_{1}l_{2}...l_{\omega}$ of length $\omega$. The probability of the transition $w\to w'$ is 0, unless $w'=l_{2}...l_{\omega}l_{\omega+1}$. Within a given window of length $L+2\omega$, let any word $w''$ of length $\omega+1$ occur $\hat{n}\left(w''\right)$ times. The posterior Dirichlet probability of the transition $w\to w'$ is

$p\left(w,w'\right)=\frac{\hat{n}\left(l_{1}...l_{\omega+1}\right)+c}{\sum_{l_{\omega+1}\in\left\{a,c,g,t\right\}} \left[\hat{n}\left(l_{1}...l_{\omega+1}\right)+c\right]}$,

where $c$ is the number of Dirichlet pseudo-counts, which for simplicity are independent of the word $l_{1}...l_{\omega+1}$. The transition probabilities determine a unique equilibrium probability distribution $\left\{\pi\left(w\right)\right\}$ satisfying $\pi\left(w'\right)=\sum_{\left(w\right)} \pi\left(w\right)p\left(w,w'\right)$.

There were 29,204 sequences in the PPR Database. We used a 3rd order Markov model, with 64 states and 256 transition probabilities to estimate, with a pseudo-count of $c=1/2$ in Eq . (The choice $c=1/2$ makes the Dirichlet prior non-informative [[4](#_ENREF_4)].) Our choice of window width for estimating the Markov transition probabilities was (the nice round number) 50 = 3 + 21 + 3 + 23, where the terms on the right have the following meanings. The Markov model was order 3 and the longest JASPAR matrices had 21 columns, leading to a decision to recalculate the Markov transition probabilities every 23 columns in the alignment. The choice of 23 columns also provides an acceptable compromise between computational speed, sensitivity to changing local nucleotide compositions, and the accuracy of the Markov transition probability estimates. For each of the 4^3^ * (4 – 1) = 192 free Markov parameters $p\left(w,w'\right)$, e.g., the Markov fit used an average of 29204 * (50 – 3) / 192 ≈ 7149 counts.

**The Implementation of the TFBS Model**: The numerator of the odds ratio in Eq corresponds to an alternative hypothesis based on the JASPAR count matrix. The following suppresses the positional subscripts in $q\left(a\right)=q_{k}\left(a_{j+k}\right)$ that appear in the numerator of the final expression in Eq . If $\left\{\hat{n}\left(a\right)\right\}$ are the empirical JASPAR counts of the nucleotides, then we estimated the target frequencies as

$\hat{q}\left(a\right)=\frac{c+\hat{n}\left(a\right)}{\sum\left[c+\hat{n}\left(a\right)\right]}$,

with pseudo-counts $c=1/2$, again making the prior probability non-informative.

**The Local Sum Statistic:** Now, let $g$ be an arbitrary parameter (which we fix later). We call $g$ the “gap penalty” because of loose analogies to the theory of gapped pairwise sequence alignment. The “global sum” $S_{i}=\sum_{j=1}^{i} \left(x_{j}-g\right)=\sum_{j=1}^{i} x_{j}-ig$ yields a “local sum” $\hat{S}_{i}=\max_{0\leq j\leq i}\left(S_{i}-S_{j}\right)$, and the local maximum $\hat{M}_{n}=\max_{0\leq i\leq n}\hat{S}_{i}$. The global sum, the local sum, and the local maximum all have analogs in sequence alignment, so our analysis used the relevant algorithms and statistics, as follows.

**Maximal Segments yield TF Motifs Clustered in Alignment Columns:** Define the (half-open) integer segment $I=\left(i,j\right]=\left\{i+1,i+2,...,j\right\}$ (a standard notation) and its score $\hat{S}\left(I\right)=S_{j}-S_{i}$. By convention, $\left(i,i\right]=\emptyset$ is permitted, and $\hat{S}\left(\emptyset\right)=0$. If $\hat{S}\left(I\right)$ is large, then $\sum_{m=i+1}^{j} x_{m}$ dominates $\left(j-i\right)g$, suggesting a large concentration of positive scores $x_{m}$ in $I$. The segment $I$ has the “Subsegment Property” iff (if and only if) $\hat{S}\left(I'\right)<\hat{S}\left(I\right)$ for every strict subsegment $I'\subset I$. A segment $I\neq\emptyset$ is “maximal” iff (1) it has the Subsegment Property (so $0<S\left(I\right)$, because $\emptyset\subset I$); and (2) there is no segment $I'\neq I$ such that $I\subset I'$ and $I'$ has the Subsegment Property. In some sense, therefore, every segment with a large concentration of positive scores $x_{m}$ is included in a maximal segment. The Ruzzo-Tompa algorithm finds all maximal segments in time $O\left(n\right)$, where our alignment has $n=3001$ columns. The details of the algorithm can be found elsewhere [[5-7](#_ENREF_5)].

**A p-value for Maximal Segments**: The following proposition is relevant.

**Proposition**: Define a random variate $Z$ whose value is uniformly distributed over the positive totals $\left\{x_{n\left(i\right)}:i=1,2,...,n'\right\}$, i.e., . Let $\nu=n'/n$, and consider a Bernoulli process $\left(n,\nu\right)$, i.e., toss $n$ weighted coins, each with head probability $\nu$. If the $i$-th coin comes up heads, associate with it a random score $X_{i}$ chosen independently from the distribution of $Z$; otherwise, let $X_{i}=0$. Given any $\rho>1$, let and consider the global sum $S_{i}=\sum_{j=1}^{i} \left(X_{i}-g\right)$ and the corresponding local sums and local maximum, defined above.

Let $\lambda>0$ be the unique positive solution of

.

Define

.

If $\mu=Ke^{-\lambda y}n$, then for $n$ large enough.

The following heuristic underlies the proposition. For $n$ large enough, a Poisson process accurately approximates the Bernoulli process. For the Poisson process, the distribution of the local maximum $\hat{M}_{n}$ is known analytically, as given above. See, e.g., [[8](#_ENREF_8)].

The Poisson process is continuous; but the Bernoulli process, discrete, so the maximal intervals $I$ suffer edge effects. To correct for the edge effects, we increased the lengths of maximal intervals by 1 and calculated the corresponding p-values conservatively, as . (Rigorous theorems can justify the inequality in the limit $n\to\infty$.)

In practice, the proposition shows that for long sequences ($n$ large), and any interval $I\subset\left(0,n\right]$

,

so any maximal segment with a score greater than $y+g$ has a p-value not exceeding $1-e^{-\mu}$.

To summarize, the Ruzzo-Tompa algorithm finds maximal segments, and the proposition above bounds the corresponding p-values conservatively.

**The Implementation of the p-value for Maximal Segments**: The proposition above has a single arbitrary parameter, $\rho$. To determine reasonable values for $\rho$, consider that various TF motifs within a cluster can have different 3’ end positions. Add one to the maximum difference between end positions, and call it the cluster’s “spread”. (Thus, a cluster has spread 1, if every TF motif in it has the same 3’ end.) In an exploratory pilot study, we collected files containing the positive scores for each TF over the PPR Database. *A priori* (as confirmed in the Results section), small files are likely to correspond to information-rich TF motifs and therefore statistically significant results. For each of the 20 smallest files, for the corresponding TFs and the plus strand of the PPR Database, computation yielded clusters within one hour. Many clusters for $\rho=1.3$, $\rho=1.4$ and $\rho=1.5$ were robust to the choice of $\rho$, with their lengths decreasing as $\rho$ increased. For $\rho=1.3$, 7 out of 25 clusters at $p\leq0.2$ had lengths 10 or more; for $\rho=1.5$, 8 out of 16 clusters at $p\leq0.2$ had spreads 1 or 2. Mindful of the possible experimental imprecision of TSS placement, and of the wish to correlate TF function with the precise positions of TF motif clusters, we compromised somewhat arbitrarily on the value $\rho=1.4$ for further intensive computation.

**DAVID** **Web Tool for Evaluating the Biological Function of a Group of Genes**: We used the DAVID Web Tool Version 6.7 at <http://david.abcc.ncifcrf.gov/> to extract annotation terms and to validate our clusters’ gene groups [[9-11](#_ENREF_9)]. DAVID has a list of biological functions (annotation terms) and for each function, a corresponding gene group. Using a (modified) Fisher Exact Test, DAVID evaluates the overlap of an input gene group with each of DAVID’s functional gene groups, inferring whether the input gene group has an associated biological function. DAVID also permits a user to specify a background set, a universe of genes under consideration, for the Fisher Exact Test. As mentioned above, after discounting alternative TSSs and alternative splices, the PPR Database corresponded to 5834 unique genes (RefSeq NP IDs). DAVID’s options were set as follows: (1) Count = 2 (i.e., display only annotations terms corresponding to at least 2 genes); (2) threshold = 0.1 (i.e., display only annotations with threshold p-value $\text{p}\leq0.1$). We report the smallest DAVID p-value for each cluster, Bonferroni-corrected by DAVID for the number of biological functions that DAVID examined.

DAVID requires gene groups as input, so for each cluster we had to map cluster sequence sets to gene groups. Each sequence was associated with both a gene from EntrezGene and a (possibly empty) set of RefSeq proteins with NP numbers. For each sequence corresponding to a fixed EntrezGene gene, the sets of RefSeq proteins became the same, after deleting the following anomalous RefSeq IDs: NP_056178, NP_065153, NP_001032824, NP_001178, NP_859055, NP_872287, NP_872290, and NP_116289. Each gene corresponded to a unique RefSeq ID in DAVID input, after we deleted all genes without a RefSeq ID and deleted all but the smallest NP number among the remaining genes’ RefSeq IDs. The resulting set of unique RefSeq IDs comprised our “DAVID Dataset”.

Our DAVID Dataset might have inherited unknown protein biases from the PPR Database. The biases could have influenced DAVID’s statistical tests, if the statistical tests in DAVID had used the full complement of human proteins as the background universe of genes. We therefore used our DAVID Dataset as the universe of genes under consideration when examining cluster functionality with DAVID and performing Fisher Exact tests for cluster overlap, next.

**Fisher Exact Tests of Intersections of Cluster Gene-Groups:** A right-tailed Fisher Exact test evaluated whether pairs of gene groups had an unusually large intersection, using our DAVID dataset as the universe of genes; a left-tailed Test, whether they had an unusually small intersection. The 2 * 43 * 42 / 2 tests require $\text{p}\leq0.05/\left(1086\right)=2.77\text{x10}^{\text{-5}}$ at significance level $\alpha=0.05$. Because the intersections are so numerous, we relegate the complete report of both the p-value from our Fisher Exact tests and the smallest DAVID p-value (as described above) for the intersections to the Supplementary Data.

**The Jaccard Distance Evaluates Overlap of Gene Group Sequences and Functions**: Let $\#A$ denote the number of elements in the set $A$. The Jaccard Distance between two sets $A$ and $B$,

$J_{\delta}\left(A,B\right)=1-\frac{\#\left(A\cap B\right)}{\#\left(A\cup B\right)}$,

is both a proper metric and a standard measure of set dissimilarity [[12](#_ENREF_12)]. The Jaccard Distance $J_{\delta}\left(A,B\right)$ can quantify the dissimilarity of the sets of sequences constituting any two gene groups $A$ and $B$. As above, subject to its thresholds, DAVID lists corresponding sets $F_{A}$ and $F_{B}$ of biological functions, so $J_{\delta}\left(F_{A},F_{B}\right)$ can quantify the functional dissimilarity of the two gene groups $A$ and $B$.

# Results

**Figure S1: Variation in (A) dinucleotide composition and (B) transposable elements content around the putative TSS**

Against the position relative to the TSS in bp on the X-axis, Figure 1A plots the fraction of the dinucleotides CpG (solid black line) and TpA (dotted grey line); Figure 1B, the fraction of sequences with a TE at the position. In both, the distinct behavior at 0 bp suggests that the PPR Dataset places many of its TSSs accurately.

**The PPR and Random Databases**: In the Supplementary Information, the file 29204_promoter.fa contains our PPR Database; the file 29204_random.fa, our Random Database. Both contained 29,204 sequences and 97,385,451 nucleotides. The PPR Database was composed of A (26.27%), T (27.73%), C (22.47%), and G (23.53%). Figure 1A shows systematic variation in base composition over the alignment columns, with spikes in C and G frequencies near the putative TSS, confirming that the anchored alignment placed putative TSSs consistently. Additionally, Figure 1B shows systematic variation in RepeatMasker repeats, with a lack of repeats near the putative TSS, again confirming consistent placement. The Random Database was composed of A (28.35%), T (28.36%), C (21.57%), and G (21.72%).

**The Measure of How Nearly a Count Matrix is a Reverse Palindrome**: To measure asymmetry of JASPAR count matrices, we computed the empirical probability distribution corresponding to each column of a count matrix. We then reversed the columns and complemented the probability distribution by mapping (A, C, G, T) to (T, G, C, A). We then computed the total variation distance between the original and final probability distributions in each column, and then averaged the result over all columns, to derive a measure of the asymmetry of a count matrix. The measure has the minimum value 0.00 if the count matrix is a perfect reverse palindrome, and it has the maximum value 1.00.

In mathematical terms, define the nucleotide alphabet . Let the count matrix have $n$ columns, and let the count for nucleotide $a$ in column $j$ of the count matrix be $c_{a,j}$ (; $j=1,2,...,n$). The empirical probability of nucleotide $a$ in column $j$ is

.

Let $\gamma$ be the complementation operator, so $\gamma\left(A\right)=T$, $\gamma\left(C\right)=G$, $\gamma\left(G\right)=C$, and $\gamma\left(T\right)=A$. Then, the probability of nucleotide $a$ in column $j$ after reversing the columns and complementing the probability distribution is

$p'_{a,j}=p_{\gamma\left(a\right),n-j+1}$.

The total variation distance between two probability distributions and is defined as

,

so if and , then the average total variation distance

$n^{-1}\sum_{j=1}^{n} d_{TV}\left(\mathbf{p}_{j},\mathbf{p}'_{j}\right)$,

measures how nearly a count matrix is a reverse palindrome.

**The Measure of the GC Content of a Count Matrix**: With notation as above, the average GC content of a count matrix is

$n^{-1}\sum_{j=1}^{n} \left(p_{C,j}+p_{G,j}\right)$.

The measure has minimum value 0.00 if the count matrix has no C or G, and it has the maximum value 1.00 if the count matrix has no A or T.

**The Measure of the Information Content of a Count Matrix**: With notation as above, the information content of a count matrix is

.

We did not correct the information content for the effect of finite samples, because we required only a crude approximation to estimate file sizes.

**Reverse Palindromic Count-Matrices:** Some JASPAR count-matrices are nearly reverse palindromic. The SI above describes a measure, under which NHLH1, STAT1, and NFKB1 (ranks 1, 2, and 3) are unusually reverse palindromic among the JASPAR count matrices. Let two clusters form a “complementary cluster-pair” if they are close to each other, one on the plus strand and one on the minus strand. Clusters for NHLH1 and NFKB1 occurred only in complementary cluster-pairs, whereas STAT1:+3:+4:+ is the only STAT1 cluster. Complementary cluster-pairs occur, however, for many other TFs (ELK4, GABPA, MYC-MAX, PPARG, RELA, RXRA-VDR, SP1, SRF, TAL1-TCF3, and TFAP2A), so complementary cluster-pairs do not require a reverse palindromic TF.

# Discussion

**Control of Artifacts in Our Study and Implications for Other TF Sequence Studies**: Our statistical methods took conservative options wherever possible, and the DAVID database [[10](#_ENREF_10), [11](#_ENREF_11)] validated our results. As in any statistical study, however, our methods only find correlations, not causality or biological activity. Our algorithms and statistical methods are computationally fast (linear in total DNA length) but do not use Monte Carlo, so they were able to handle genomic-scale datasets deterministically. Our techniques therefore permit some tentative observations about the relationship between TF motifs, functional TFBSs, and sequence biases.

First, background composition can cause false positive TFBS predictions. Here, however, an unusually detailed Markov model (order 3) controlled for background composition. To account for systematic compositional variations due to DNA isochores, we even recomputed empirical Markov transition probabilities within a window of width 50 bp every 24 bp across a block alignment. Compositional controls were therefore unusually elaborate, but nonetheless, all clusters with broad spreads (for E2F1, ELK4, RREB1, and SP1) still reflected the GC compositional bias of proximal promoters. DAVID still strongly validated all broad clusters, however, suggesting that the corresponding TFBSs have adapted themselves to reflect the necessary compositional biases.

Unlike the present study, many computational TFBS studies rely purely on sequence. If they maintain tight bounds on type I error by compensating for background compositional biases as stringently as we did, they probably severely reduce their power to locate GC-biased TFBSs.

Second, nucleotide composition swings sharply near the TSS. (See Figure 1A in the SI.) Typically, background models are homogeneous over a window, so they cannot control for such localized compositional variation. Indeed, compositional bias at the TSS appears problematic in any sequence study. Validation provides some remedy, but the issue of statistical correlation versus causality renders it a partial panacea.

Third, DNA tandem repeats and low-complexity sequences violate background Markov models [[13](#_ENREF_13)]. We were reluctant to mask them, however, because when part of a transposable element [[14-18](#_ENREF_14)], they can enhance TF binding. Fortunately, the Results section effectively excludes repetitive artifacts. Again, however, if a study relying exclusively on sequence were to maintain tight bounds on type I error by masking repetitive elements, it probably would severely reduce its power to locate TFBSs.

DAVID’s validation is also subject to biases. Our PPR Database originates with expression data, yielding a possible bias toward highly expressed genes that DAVID might share. Against a universe of all human genes, therefore, DAVID validation of our results might only reflect common biases toward highly expressed genes. (Even normalized, microarray expression data probably have worse expression biases than DAVID for validating our results.) To defend against any over-representation of specific human genes in our PPR Database, our Fisher Exact tests and validation with DAVID used our DAVID Dataset (all genes in significant clusters) as its universe.

**The Search for a Single TF with Two Clusters Having Antagonistic Functions**: By analogy to chemical similarities between receptor ligands and their antagonists, the possibility that two clusters for a single TF might have antagonistic functions intrigued us. We therefore examined pairs of motif clusters where both clusters correspond to a single TF. For all such pairs, we looked for a trend between the physical DNA distance separating the cluster-pairs and the Jaccard distance between the corresponding pairs of gene groups. Here, a large Jaccard distance indicates that the gene groups are mostly disjoint, suggesting different biological functions. The file clusters.xlsx in the SI shows that in fact, cluster-pairs proximal to each other often had small Jaccard distances, suggesting that they corresponded to a single TFBS cluster that our statistical methods had incorrectly partitioned into two or more. Except for the trend in proximal cluster-pairs, we found neither a trend nor any obvious outliers when we plotted physical distance and Jaccard distance between: (1) pairs of gene groups; (2) the corresponding list of DAVID functional terms for pairs of gene groups, and (3) the corresponding list of DAVID functional clusters for pairs of gene groups.

We also searched cluster-pairs with validating FDRs less than 0.2 whose annotation terms in DAVID had a common stem prefaced by “positive” for one cluster and “negative” for the other, but found none. If TFBSs in different positions have antagonistic functions, our study was unable to resolve them.

**Possible Artefacts Enriching Gene Groups Corresponding to the Intersections of Significant Cluster-Pairs**: The scarcity of *p*-values $\text{p}\leq0.20$ among the 903 Fisher exact left-sided *p*-values suggests an influence, biological or artefactual, subtly enriching gene groups corresponding to intersections of significant cluster-pairs.

Among biological influences, perhaps some TFBS cluster-pairs with nearby binding sites contribute to antagonistic submodules (since the TFBSs co-occur, the corresponding TFs cannot bind to the same DNA simultaneously). As described above, however, we searched unsuccessfully for antagonistic submodules. Alternatively, the Absolutely Positioned Distant Submodule Hypothesis posits that more than a scattered few of the 903 pairs of motif clusters actually do co-occur in at least one co-regulating CRM architecture, systematically enriching the corresponding intersections. Unfortunately, as the Discussion section in the article states, our Results do not support the Absolutely Positioned Distant Submodule Hypothesis.

Among artefacts, composition might enrich intersections within disjoint classes of TFBSs (e.g., AT-rich TFBSs, GC-rich TFBSs), but seems unlikely to enrich nearly all intersections. By itself, gene over-expression does not bias the Fisher Exact test away from its intended meaning, because each gene is simply present or absent in each TF motif cluster. Some TF motifs in significant clusters are false positives and do not correspond to TFBSs. If the false positives occur randomly, they correspond more frequently to genes over-represented among the significant clusters than to other genes. Moreover, such gene over-representation probably occurs, because: (1) the Database of Transcriptional Start Sites (DBTSS) favours highly expressed genes; and (2) our PPR Database contains multiple copies of each gene, one copy for each alternative TSSs from DBTSS.

**References**

1. Kim NK, Tharakaraman K, Spouge JL: **Adding sequence context to a Markov background model improves the identification of regulatory elements**. *Bioinformatics* 2006, **22**(23):2870-2875.

2. Liu X, Brutlag DL, Liu JS: **BioProspector: discovering conserved DNA motifs in upstream regulatory regions of co-expressed genes**. *Pac Symp Biocomput* 2001:127-138.

3. Thijs G, Lescot M, Marchal K, Rombauts S, De Moor B, Rouze P, Moreau Y: **A higher-order background model improves the detection of promoter regulatory elements by Gibbs sampling**. *Bioinformatics* 2001, **17**(12):1113-1122.

4. Jeffreys H: **Theory of Probability**, 3 edn. Oxford: Oxford University Press; 1961.

5. Ruzzo WL, Tompa M: **A linear time algorithm for finding all maximal scoring subsequences**. *Proc Int Conf Intell Syst Mol Biol* 1999:234-241.

6. Spouge JL, Marino-Ramirez L, Sheetlin SL: **The Ruzzo-Tompa algorithm can find the maximal paths in weighted, directed graphs on a one-dimensional lattice** In: *Computational Advances in Bio and Medical Sciences (ICCABS), 2012 IEEE 2nd International Conference on: 2012; Las Vegas*. IEEE Xplore.

7. Spouge JL, Marino-Ramirez L, Sheetlin SL: **Searching for repeats, as an example of using the generalised Ruzzo-Tompa algorithm to find optimal subsequences with gaps**. *International Journal of Bioinformatics Research and Applications* 2014, **10**(4):384-408.

8. Frith MC, Spouge JL, Hansen U, Weng Z: **Statistical Significance of Clusters of Motifs Represented by Position Specific Scoring Matrices in Nucleotide Sequences**. *Nucleic Acids Res* 2002, **30**(14):3214-3224.

9. Huang D-W, Sherman BT, Lempicki RA: **Bioinformatics enrichment tools: paths toward the comprehensive functional analysis of large gene lists**. *Nucleic Acids Res* 2009, **37**(1):1-13.

10. Dennis G, Sherman BT, Hosack DA, Yang J, Gao W, Lane HC, Lempicki RA: **DAVID: Database for annotation, visualization, and integrated discovery**. *Genome Biol* 2003, **4**(9).

11. Huang D-W, Sherman BT, Lempicki RA: **Systematic and integrative analysis of large gene lists using DAVID bioinformatics resources**. *Nat Protoc* 2009, **4**(1):44-57.

12. Jaccard P: **Étude comparative de la distribution florale dans une portion des Alpes et des Jura**. *Bulletin de la Société Vaudoise des Sciences Naturelles* 1901, **37**: 547–579.

13. Davis IW, Benninger C, Benfey PN, Elich T: **POWRS: Position-Sensitive Motif Discovery**. *PLoS ONE* 2012, **7**(7).

14. Wang J, Bowen NJ, Marino-Ramirez L, Jordan IK: **A c-Myc regulatory subnetwork from human transposable element sequences**. *Mol Biosyst* 2009, **5**(12):1831-1839.

15. Polavarapu N, Marino-Ramirez L, Landsman D, McDonald JF, Jordan IK: **Evolutionary rates and patterns for human transcription factor binding sites derived from repetitive DNA**. *BMC Genomics* 2008, **9**:226.

16. Huda A, Marino-Ramirez L, Landsman D, Jordan IK: **Repetitive DNA elements, nucleosome binding and human gene expression**. *Gene* 2009, **436**(1-2):12-22.

17. Marino-Ramirez L, Jordan IK: **Transposable element derived DNaseI-hypersensitive sites in the human genome**. *Biol Direct* 2006, **1**.

18. Huda A, Marino-Ramirez L, Jordan IK: **Epigenetic histone modifications of human transposable elements: genome defense versus exaptation**. *Mob DNA* 2010, **1**(1):2.
